# LLM-as-a-judge for infection prevention and control and antimicrobial resistance impact: comparing three main LLMs vs. human experts' assessment

**DOI:** 10.3389/fpubh.2026.1874389

**Published:** 2026-07-14

**Authors:** Marcello Di Pumpo, Leonardo Villani, Maria Rosaria Gualano, Danilo Buonsenso, Francesca Raffaelli, Daniele Donà, Patrizia Laurenti, Vittorio Maio, Stefania Boccia, Walter Ricciardi

**Affiliations:** 1 Section of Hygiene, University Department of Life Science and Public Health, Università Cattolica del Sacro Cuore, Rome, Italy; 2 Italian Society for Artificial Intelligence in Medicine (SIIAM—Società Italiana Intelligenza Artificiale in Medicina), Rome, Italy; 3 UniCamillus—Saint Camillus International University of Health and Medical Sciences, Rome, Italy; 4 Department of Woman and Child Health, Fondazione Policlinico ‘Agostino Gemelli' IRCCS, Rome, Italy; 5 Area Pediatrica, Dipartimento di Scienze della Vita e Sanità Pubblica, Università Cattolica del Sacro Cuore, Rome, Italia; 6 Department of Laboratory and Infectivology Sciences, Fondazione Policlinico Universitario A. Gemelli IRCCS, Rome, Italy; 7 Department of Women's and Children's Health, University of Padova, Padua, Italy; 8 Jefferson College of Population Health, Thomas Jefferson University, Philadelphia, PA, United States; 9 Asano-Gonnella Center for Research in Medical Education and Health Care, Sidney Kimmel Medical College, Thomas Jefferson University, Philadelphia, PA, United States

**Keywords:** antimicrobial resistance (AMR), large language models (LLM), LLM-as-a-judge, LLMs rating, public health

## Abstract

**Background:**

Large language models (LLMs) are increasingly used to generate health information, yet their reliability as evaluators remains unclear. This study investigated the feasibility of an LLM-as-a-judge methodology in the context of infection prevention and antimicrobial resistance (AMR), comparing automated ratings with human expert benchmarks.

**Methods:**

We performed a secondary analysis of an expert-annotated dataset of health messages. Three leading LLMs (ChatGPT, Claude, Gemini) independently evaluated the same messages using an adapted DISCERN tool across five domains: information reliability, quality, AMR impact, persuasiveness, and overall score. We utilized descriptive statistics, intra-rater reliability tests, and mixed-effects ordinal regression to analyze divergence between automated and human assessments, adhering to CHART reporting guidelines.

**Results:**

Analysis of 404 evaluations revealed a systematic upward divergence: all LLMs consistently assigned higher scores than human experts. This optimism bias persisted after adjusting for domain-specific differences and clustering effects. The gap was particularly pronounced in domains of persuasiveness and AMR impact, while information quality showed more heterogeneous results. Intra-rater reliability assessments demonstrated that LLMs maintained stable scoring patterns under identical prompting conditions.

**Conclusions:**

LLMs exhibit a consistent leniency bias, systematically overestimating the quality of AMR-related health communication compared to human evaluators. These results do not support the use of LLMs for autonomous evaluation in high-stakes public health contexts. Rather, LLM-based judging is best suited as a scalable screening tool within supervised human-in-the-loop workflows, where expert oversight serves as a necessary safeguard for evidence-based accuracy.

## Introduction

Large language models (LLMs) are increasingly used to generate health-related informational and promotional texts, offering rapid, low-cost drafting alongside fluent language generation (1). Recent studies indicate that while LLMs can efficiently produce public health messages across topics and languages, the quality, clarity, and potential risk of these outputs vary substantially (2–4). As a result, the increasing automation of text generation in health communication workflows has positioned content evaluation as a critical bottleneck.

Expert review remains fundamental for validating materials that influence health behaviors, risk perceptions, and institutional trust. Such evaluation extends beyond linguistic fluency to encompass appropriateness, potential harm, and interpretive nuance. However, the resource-intensive nature of expert assessment creates structural challenge for scalability. To address this imbalance, recent research in Natural Language Processing (NLP) has explored the use of LLMs not only as generators but also as evaluators- or “judges”- of text quality (5).

Despite early promise, notable limitations persist. Correlation-based evaluation metrics can be misleading in the presence of variability in human judgments, and commonly used benchmarks often fail to account for inherent rating indeterminacy, thereby inflating estimates of agreement between automatic judges and human annotators (6–9).

Within health-related contexts, evidence regarding agreement between LLM-based evaluators and domain specialists remains mixed. Some studies report only moderate convergence between LLM assessments and expert judgments in specialized tasks, underscoring the importance of domain expertise. Conversely, in structured settings, such as rubric-guided evaluation of clinical summaries, LLM judgments can approach meaningful levels of agreement when assessed using formal reliability metrics (3, 4, 10–12). Taken together, these findings suggest that automated evaluation may be viable in health settings when tasks are clearly specified and human oversight is maintained (13).

In real-world public health workflows, messaging is typically developed through iterative cycles of generation and refinement. In this context, LLMs could serve as high-sensitivity screening tools to prioritize content for expert review within human-in-the-loop (HITL) frameworks, thereby reducing workload while preserving safety and quality oversight.

Building on these developments, the present study examines the feasibility and validity of using widely available LLMs as evaluators of public health communication messages on antimicrobial resistance (AMR), previously assessed by domain experts using a DISCERN-adapted instrument.

## Methods

### Study design

We conducted a comparative secondary analysis using a previously published dataset of LLM-generated health information messages that had been independently rated by human experts. In the parent study (14), healthcare expert–designed scenarios were submitted to multiple LLMs, and the resulting outputs were evaluated by three domain experts using structured ordinal rating criteria. This process yielded a comprehensive set of item-level domain scores across two languages (English and Italian).

In the present study, previously collected human expert ratings were used as an operational reference benchmark against which automated LLM-based evaluations were compared. The purpose was not to establish an objective ground truth, but rather to examine the degree to which contemporary LLM evaluators align or diverge from structured assessments produced by domain experts under a standardized rating framework. Given the inherently interpretive nature of evaluative judgment tasks, expert ratings were considered a pragmatic comparative reference rather than a definitive gold standard. To characterize the consistency of this benchmark, agreement among the three human expert raters was assessed using pairwise quadratic-weighted Gwet's AC2 coefficients. Overall median agreement was 0.62, with domain-specific coefficients ranging from 0.61 for overall score (D5) to 0.74 for information quality (D2), while agreement across individual rater pairs varied from 0.51 to 0.81. This degree of variability was considered consistent with the expected heterogeneity of expert-based evaluative judgment tasks, in which legitimate differences in interpretation may occur even when raters apply the same structured criteria.

Specifically, we analyzed messages previously generated by LLMs and evaluated by human experts, and we tasked contemporary LLMs with assigning ordinal quality scores to the same texts.

The LLM that originally generated each message was included as a structural covariate for analytical control and was not interpreted as a proxy for message quality. This design enabled disentanglement of generator effects from that rater effects. Furthermore, the use of crossed random-effects models allowed separate estimation of variability attributable to generation vs. rating process. Evaluation domains were based on an adapted DISCERN instrument, focusing on fine dimensions: reliability of information, quality of information, contribution to addressing AMR, persuasiveness, and overall quality score.

### Large language models selection and configuration

Three state-of-the-art proprietary LLMs were selected as automated raters: OpenAI ChatGPT−5.2 (December 2025), Google DeepMind Gemini 3 Pro (November 2025), and Anthropic Claude Sonnet 4.6 (February 2026), as available at the time and place of the study. All models were closed-source base systems, and no fine-tuning or modification was performed by the investigators.

The LLMs that generated the original messages in the parent study and underwent human rating were treated solely as structural covariates. Rater LLMs were accessed on February 28, 2026, from Padua, Italy, via each provider's official web-based consumer interface, using subscription-based personal accounts. No application programming interface (API) access, custom code, or explicit hyperparameter tuning was employed.

This approach was intentionally chosen to reflect real-world usage patterns, as web-based interfaces represent the most common mode of interaction and thus the context with the greatest practical impact. However, prompts explicitly instructed models to prioritize reproducibility and deterministic behavior (e.g., minimizing temperature and requesting consistent outputs across runs).

### Prompting strategy and score generation

A standardized prompt, derived from the adapted DISCERN instrument (Supplementary File S1) was developed by two researchers (MDP, LV) and tailored for each LLM. A one-shot prompting strategy was adopted, requiring each model to return all domain scores in a single structured response.

Outputs were specified using 5-point Likert scale (1=Poor, 2=Suboptimal, 3=Fair, 4=Good, 5= Excellent). To assess intra-rater reliability, 10 independent iterations were conducted per model under identical conditions. Each iteration was initiated in a new browser session with no prior conversational history, thereby minimizing context carryover and memory effects.

Rater LLMs received only anonymised message text and the scoring rubric. No additional metadata, such as generator identity, item order, or human expert ratings, was provided. Prompts were identical across all evaluations, with only the message content varying. Each response consisted of a structured set of five ordinal domain scores.

No patient or public involvement was included in the evaluation process. The sample size corresponded to the full dataset from the parent study, and no formal *a priori* power calculation was conducted. All prompts are available in Supplementary File S1.

### Statistical analysis

Descriptive statistics were computed according to variable type and distribution. Normality was assessed using the Shapiro-Wilk test. Human inter-rater and LLM intra-rater agreements were estimated using quadratic-weighted Gwet's AC2 (15), selected for its robustness in ordinal data and resistance to prevalence and bias effects. For inferential analysis, a Friedman test was employed as a nonparametric approach to assess differences across raters. Ordinal logistic regression models were then fitted to estimate associations between rater type and assigned scores.

A baseline crude logistic regression model including only rater LLM as a predictor was first specified. Subsequently, an adjusted mixed-effects ordinal logistic model was fitted, including rater LLM and evaluation domain as fixed effects, with random intercepts for message ID (to account for repeated measurements of the same text) and generator LLM (to account for clustering by the original generating model). A sensitivity analysis based on dichotomized ratings was performed to ensure the robustness of findings from the ordinal outcomes analyses. Variance decomposition and language-stratified analysis were also performed on the adjusted fitted regression models.

All analyses were conducted using R version 4.5.0 (R Core Team, 2025).

### Ethics consideration and reporting

This study constitutes a secondary analysis of previously published, de-identified ordinal rating data, with no use of patient-identifiable information. Ethical approval was therefore not required. The study adhered to the CHART reporting guideline for chatbot health advice studies (16). A completed CHART checklist is provided in Supplementary File S2.

## Results

A total of 404 ratings were analyzed, consisting of 101 evaluations for each of the three LLM raters (ChatGPT, Claude, and Gemini) and 101 baseline human evaluations.

The Shapiro–Wilk test rejected the null hypothesis of normality (*p* < 0.001), warranting the use of non-parametric statistics.

Across all observations (*n* = 404), systematic differences emerged between human and LLM raters (Table 1). Human evaluators assigned lower scores overall (median total score = 16, IQR: 13–17; median mean-item score = 3.2), whereas all LLM raters showed higher central tendencies. ChatGPT exhibited the highest scoring pattern (median total = 19, IQR: 15–21; median mean-item = 3.8), followed by Claude and Gemini (both median total = 18; median mean-item = 3.6). These descriptive findings suggest a consistent upward shift in LLM-based evaluations relative to human judgment.

**Table 1:** Descriptive statistics by rater.

Rater	*n*	Median (IQR)
Human rating	101	16 (13-17)
Claude	101	18 (17-19)
Gemini	101	18 (17-20)
ChatGPT	101	19 (15-21)

Regarding consistency across repeated prompting sessions, intra-LLM agreement was assessed using Gwet's AC2. Agreement coefficients were 0.84 for ChatGPT, 0.68 for Claude, and 0.77 for Gemini, indicating substantial agreement across prompt repetitions for all three models, although full reproducibility was not achieved.

In the crude ordinal logistic regression (Table 2), all LLM raters were significantly more likely than human evaluators to assign higher scores. Gemini showed the strongest divergence (OR 3.92; 95% CI 3.09–4.99; *p* < 0.001), followed by ChatGPT (OR 3.07; 95% CI 2.43–3.88; *p* < 0.001) and Claude (OR 2.83; 95% CI 2.25–3.55; *p* < 0.001). These results indicate that, without accounting for structural factors, LLMs consistently overestimate quality relative to human raters. After adjusting for domain and accounting for clustering at the item ID and generator LLMs, the upward trend remained and increased in magnitude (Table 3). Gemini exhibited the strongest adjusted effect (OR 5.73; 95% CI 4.46–7.37; *p* < 0.001), followed by ChatGPT (OR 4.35; 95% CI 3.39–5.58; *p* < 0.001) and Claude (OR 3.92; 95% CI 3.08–4.99; *p* < 0.001). The amplification of odds ratios after adjustment suggests that part of the crude variability was masking an even stronger systematic divergence between LLM and human ratings.

**Table 2:** Crude ordinal logistic model.

Rater	OR (CI 95%)	*P* value
Claude	2.83 (2.25–3.55)	< 0.001
Gemini	3.92 (3.09–4.99)	< 0.001
ChatGPT	3.07 (2.43–3.88)	< 0.001

Human expert ratings were the reference category. Odds ratios are relative to human ratings.

**Table 3:** Adjusted mixed-effects ordinal logistic regression results.

Rater	OR (CI 95%)	*P* value
Claude	3.92 (3.08–4.99)	< 0.001
Gemini	5.73 (4.46–7.37)	< 0.001
ChatGPT	4.35 (3.39–5.58)	< 0.001

Human expert ratings were the reference category. Odds ratios are relative to human ratings.

Domain-specific models confirmed that LLM–human divergence varied substantially across domains (Table 4). The strongest upward bias emerged for contribution to addressing AMR (D4), where all LLMs were significantly more likely than human experts to assign higher scores: Claude, OR = 47.27 (95% CI: 23.34–95.75; *p* < 0.001); Gemini, OR = 83.35 (95% CI: 39.28–176.87; *p* < 0.001); and ChatGPT, OR = 65.59 (95% CI: 31.47–136.72; *p* < 0.001). A similarly large divergence was observed for persuasiveness (D3), but only for Claude and Gemini: Claude, OR = 45.51 (95% CI: 14.26–145.30; *p* < 0.001), and Gemini, OR = 67.40 (95% CI: 20.24–224.47; *p* < 0.001), whereas ChatGPT did not differ significantly from human raters. For the overall rating (D5), all LLMs again assigned significantly higher scores, with the largest effect for Gemini, OR = 27.46 (95% CI: 14.05–53.67; *p* < 0.001). In contrast, information quality (D2) showed an inverse or null pattern, with Claude and Gemini significantly less likely to assign higher scores than human experts: Claude, OR = 0.51 (95% CI: 0.30–0.87; *p* = 0.013), and Gemini, OR = 0.40 (95% CI: 0.23–0.69; *p* = 0.001), while ChatGPT showed no significant difference. Table 5 reports the distribution of Likert ratings assigned by LLMs and human experts. The stratification by domain is reported in Supplementary File 3.

**Table 4:** Domain-specific ordinal logistic models: ORs for LLMs vs. human raters.

Domain	Rater	OR	CI (95%) low	*P* value
D1	Claude	10.22	5.64–18.54	< 0.001
D1	Gemini	2.37	1.36–4.12	0.002
D1	ChatGPT	12.38	6.56–23.33	< 0.001
D2	Claude	0.51	0.3–0.87	0.013
D2	Gemini	0.4	0.23–0.69	0.001
D2	ChatGPT	0.79	0.45–1.4	0.421
D3	Claude	45.51	14.26–145.3	< 0.001
D3	Gemini	67.4	20.24–224.47	< 0.001
D3	ChatGPT	0.79	0.43–1.46	0.457
D4	Claude	47.27	23.34–95.75	< 0.001
D4	Gemini	83.35	39.28–176.87	< 0.001
D4	ChatGPT	65.59	31.47–136.72	< 0.001
D5	Claude	2.22	1.32–3.74	0.003
D5	Gemini	27.46	14.05–53.67	< 0.001
D5	ChatGPT	7.95	4.36–14.48	< 0.001

D1: reliability of information, D2: quality of information, D3: persuasiveness, D4: contribution to addressing AMR, D5: overall rating score.

**Table 5:** Distribution of Likert ratings assigned by LLMs and human experts.

Rater	Score 1	Score 2	Score 3	Score 4	Score 5	Total
Claude	0 (0.0%)	16 (3.2%)	196 (38.8%)	251 (49.7%)	42 (8.3%)	505
Gemini	0 (0.0%)	54 (10.7%)	134 (26.5%)	205 (40.6%)	112 (22.2%)	505
ChatGPT	7 (1.4%)	55 (10.9%)	135 (26.7%)	234 (46.3%)	74 (14.7%)	505
Human	59 (11.7%)	70 (13.9%)	192 (38.0%)	167 (33.1%)	17 (3.4%)	505

The variance decomposition analysis showed random-intercept variances of 0.616 for message ID and 0.163 for generator LLM. On the latent logistic scale, the corresponding Intraclass Correlation Coefficients were 0.151 and 0.040, respectively. These findings indicate that approximately 15.1% of residual variability was attributable to differences between messages, whereas 4.0% was attributable to differences between LLM generators. Overall, this suggests that variability in the specific message being evaluated contributed more substantially to rating variation than the generator itself. A separate language-stratified analysis was conducted after excluding Google Gemini and adjusting for rater, domain, message ID, and generator LLM. In the language-adjusted model, there was no evidence of a statistically significant association between message language and ratings. Specifically, English-language messages showed similar odds of receiving higher ratings compared with Italian-language messages (OR = 1.05, 95% CI 0.41–2.73; *p* = 0.916), indicating no meaningful difference attributable to language.

No rater LLM produced offensive or harmful messages, refusals nor safety disclaimers; all responses conformed to the requested structured ordinal format.

## Discussion

This study compared three large language models (LLMs) with expert raters in evaluating public health messages, finding that LLMs consistently assigned higher scores. LLM ratings were stable across prompting sessions, indicating limited stochastic variability. Mixed-effects ordinal models confirmed that LLMs were significantly more likely than experts to provide higher ratings, even after accounting for clustering. The magnitude and consistency of this divergence suggest a systematic optimism bias rather than random disagreement. In particular, the large odds ratios observed in some domain-specific analyses should be interpreted cautiously. Sparse and occasionally empty score categories were present in several domain-level comparisons, which may have amplified effect estimates and widened confidence intervals. Accordingly, the direction and consistency of divergence from human ratings are likely more informative than the exact magnitude of individual odds-ratio estimates. The observed patterns therefore support conclusions regarding systematic differences in scoring behavior, while the magnitude of specific domain-level estimates should not be overinterpreted. In addition, a sensitivity analysis based on dichotomized ratings produced findings that were directionally consistent with the primary analyses, supporting the robustness of the observed patterns despite the presence of sparse score distributions.

Human expert agreement, although substantial, did not achieve full concordance, a finding that should not be interpreted as a methodological weakness unique to the present study. Unlike measurement tasks involving a single objectively correct latent value (for example blood pressure or temperature estimation), evaluative judgment tasks inherently involve interpretation. When assessing the quality or appropriateness of a health communication message, experts may reasonably differ because they assign different weight to equally legitimate dimensions such as clarity, contextual appropriateness, ethical acceptability, actionability, or scientific completeness. Importantly, even complete agreement among human raters would not transform such evaluations into an objective ground truth. Accordingly, in the present study expert ratings should be interpreted as a structured comparative benchmark rather than a definitive standard of correctness. The observed LLM-human divergence therefore reflects differences in evaluative calibration relative to expert judgment, rather than direct evidence that either evaluation framework is intrinsically correct or incorrect.

Human–LLM disagreement may instead arise as LLMs may rely more heavily on surface-level textual fluency, apply different implicit thresholds for rating categories, over-weight formal completeness or persuasiveness, or fail to incorporate contextual and disciplinary knowledge in the same way as human experts. This interpretation is consistent with the broader LLM-as-judge literature, which emphasizes that LLM evaluators can show systematic biases, prompt sensitivity, scale effects, and imperfect alignment with human judgement. In our study, the presence of both substantial human–human agreement and measurable expert disagreement supports a cautious interpretation: LLMs may provide useful complementary evaluations, but their ratings should not be treated as substitutes for expert judgement (17).

Multiple studies have documented that LLM judges exhibit pervasive leniency, verbosity preference, and positivity biases that inflate absolute scores relative to human consensus (9, 12, 13, 18). Our results extend these observations to the domain of infection prevention control and AMR, further confirming that the phenomenon is neither vendor-specific nor confined to general-purpose tasks.

A plausible mechanistic explanation for this upward shift lies in the preference-based post-training pipelines used to align current LLMs. Reinforcement learning from human feedback (RLHF) optimizes models against reward signals derived from human preferences, and there is now robust evidence that such signals encode an “agreement is good” heuristic that rewards affirming, enthusiastic, and superficially fluent responses over critical or contrarian ones (19–21). Some authors formally characterized this tendency as “sycophancy” (19, 21) as a feedback-positivity bias: when asked to rate the quality of a text, models trained to please users tend to err on the side of generous appraisal. In the medical domain specifically, a recent work by Chen et al. (22) has shown that this helpfulness-driven bias compromises factual accuracy, with frontier LLMs complying with logically flawed clinical premises up to 100% of the time in the absence of explicit rejection prompts. The upward rating shift observed in our study is not an incidental artifact but a predictable downstream consequence of current alignment paradigms.

Interesting findings emerged from domain-stratified analyses, which revealed that the LLM-human gap is highly heterogeneous across the five DISCERN-adapted dimensions evaluated.

The Persuasiveness Domain showed one of the largest divergences, but only for Claude and Gemini, while ChatGPT did not significantly differ from human raters. This suggests that higher LLM scoring in this domain was not a uniform feature of automated evaluation, but may reflect model-specific sensitivity to fluent or rhetorically polished content. This pattern is consistent with previously described style-over-substance or verbosity bias (13, 17, 22), although its practical meaning should be interpreted cautiously, particularly in infection prevention and antimicrobial use contexts where persuasive but inaccurate information may affect adherence to recommendations (23). A more consistent upward pattern was observed for Contribution to addressing AMR, where all LLMs assigned substantially higher scores than human experts, suggesting a possible tendency to overestimate the likely public health impact of health communication messages. By contrast, Information Quality showed the opposite pattern for Claude and Gemini, which were less likely than human raters to assign higher scores, while ChatGPT showed no significant difference. This indicates that LLM–human divergence was not uniformly optimistic across all domains and may differ when models assess technical or evidence-based content, in line with concerns that LLM-generated or LLM-evaluated health information may not consistently meet evidence-based communication standards (26). For Information Reliability, positive deviations were observed, especially for ChatGPT and Claude, whereas the Overall Judgment Domain showed higher ratings for all LLMs, with the largest effect for Gemini, supporting the view that global or composite judgments may amplify positivity bias compared with more specific criteria (9, 15).

The reverse pattern observed for the Information Quality domain suggests that LLM–human disagreement was not uniform in direction. However, even though Claude and Gemini were less likely than human experts to assign higher scores for information quality, this may not be interpreted as evidence that those LLM judge differently regarding technical evidence quality. The adapted DISCERN domains are in fact conceptually and psychometrically interdependent: D2 includes not only formal evidence-quality criteria, but also clarity, completeness, balance, transparency, usefulness, and support for decision-making, while dimensions such as persuasiveness, perceived AMR impact, and overall usefulness may also depend on factual adequacy and credibility. A plausible interpretation is that Claude and Gemini penalized certain information-quality features more strongly than human experts, such as absent citations, lack of explicit evidentiary markers, or limited discussion of uncertainty. However, this remains a hypothesis and may instead reflect stricter formal pattern recognition, different rating-scale calibration, or model-specific sensitivity to surface markers of evidence quality.

This has direct implications for deployment, since whether a model is more stringent or more lenient may determine where it can be responsibly integrated into human-in-the-loop assessment workflows (23).

Overall, although the domain-specific pattern points to a tendency toward higher LLM scoring, the heterogeneity across raters and the reversed findings for Information Quality argue against interpreting individual odds ratios in isolation. The variance decomposition analysis indicated that LLM raters were mostly indifferent to generator identity when assigning scores, with rating variability being driven more by the specific message being evaluated than by the model that generated it. Thus, differences among generated messages appeared more important for rating outcomes than systematic differences attributable to the generator LLM itself. The language-adjusted analysis showed no evidence that ratings differed between English and Italian messages. This finding should be interpreted in light of prior evidence showing that LLM performance may vary across languages, with English-centric advantages reported in several multilingual evaluations, particularly for low-resource or underrepresented languages (24, 25). However, language effects appear to depend on the model, task type, prompt format, and evaluation design (25, 26). In our study, the task was a constrained ordinal evaluation of health communication messages rather than open-ended reasoning, free-text generation, or question answering, which may have attenuated language-related differences. Moreover, Italian is generally considered a high-resource language in NLP research, although less represented than English in native LLM benchmarks (26). This may explain why no measurable English–Italian difference emerged in our analysis. Future studies may benefit from retrieval-grounded or reference-anchored evaluation frameworks that explicitly link LLM judgments to predefined evidence sources or evaluation criteria, potentially improving calibration and interpretability of LLM-based evaluators (27).

From an applied perspective, these findings support a supervised human-in-the-loop role for LLMs in AMR communication assessment, in which models can provide a first-pass screening and highlight cases for expert review, while final adjudication remains with human raters. Borderline, discordant, or domain-sensitive outputs should be escalated, and any disagreement between automated and human judgments should be resolved through secondary human review. Thresholds for escalation would need prospective calibration against expert consensus and local workflow constraints.

Human-in-the-loop approaches are in fact key for the governance of AI systems in healthcare (28–30), where automated scoring may support, without replacing, expert judgment.

### Strengths and limitations

To our knowledge this study among the first to apply an LLM-as-judge approach in public health research, and the first specifically focused on infection prevention and control, and AMR. It leverages a previously published, expert-annotated dataset of health promotion messages, enabling a rigorous secondary analysis with expert ratings as a structured comparative benchmark, thereby strengthening interpretability of agreement patterns. The analysis was conducted both overall and at domain level, allowing assessment of differences across subtopics and semantic evaluation dimensions. The study adhered to CHART reporting guidelines, enhancing transparency and reproducibility.

The choice to evaluate LLMs as raters through their consumer-facing interfaces allows to capture their performance in the mode of access most likely to be used by scientific researchers without specific technical expertise, providing evidence on a realistic and highly accessible use scenario.

Limitations include the dependence on the original dataset's design, content, and rating criteria, which constrains flexibility and frames expert ratings as an operational reference rather than a definitive ground truth. The dataset also covers a limited range of topics, languages, and formats, potentially limiting generalizability. Additionally, LLM raters were evaluated via standard web interfaces using one-shot prompting, reflecting platform-level behavior rather than fully controlled model properties; performance may vary under different prompting strategies or model versions. However, this approach mirrors real-world usage by non-technical users. To mitigate variability, a metaprompting strategy was applied, intra-rater reliability was assessed showing substantial agreement, and mixed-effects ordinal regression models were used to account for key sources of variability.

Considering how all LLM assessments in this study were conducted through publicly accessible web chat interfaces, the findings should be interpreted as reflecting consumer-interface behaviour rather than the performance of models deployed through application programming interfaces, locally hosted systems, or task-specific fine-tuned configurations. This distinction is important because a scalable screening tool would likely be implemented through API-based or otherwise controlled deployment pipelines, where model versioning, sampling parameters, prompt management, batching, monitoring, and domain-specific calibration could differ substantially from the conditions evaluated here. Therefore, the observed performance should not be assumed to generalize directly to production-grade or fine-tuned LLM screening systems without further validation in those deployment contexts.

For this reason, a replication via code, hyperparameters control, and API access would be feasible and methodologically valuable. Such an analysis would provide a more deployment-relevant assessment. We therefore consider this a feasible and important direction for future validation work. An additional limitation concerns the use of human expert ratings as the comparative benchmark. Although expert-based evaluation remains the most commonly adopted approach for assessing qualitative characteristics of health communication materials, such ratings do not constitute an objective ground truth. Evaluative tasks of this type are inherently interpretive, and different experts may legitimately assign different scores while applying equally valid judgment criteria. Accordingly, the observed divergence between LLM and human ratings should not be interpreted as evidence that LLM evaluations are objectively incorrect, but rather that they follow a systematically different evaluative calibration relative to domain expert judgment. The moderate inter-rater agreement observed among human experts reflects this inherent variability and represents a general limitation of human-centered benchmarking approaches in this field.

## Conclusion

The study found that LLMs tend to exhibit systematic upward divergence from expert judgments when used via an LLM-as-a-judge methodology. The magnitude and stability of this effect across vendors support the conclusion that the divergence reflects a general calibration difference rooted in current alignment paradigms and, potentially, the web interface, rather than an idiosyncratic behaviour of any single model. The findings do not support autonomous LLM-based appraisal of public health communication in high-stakes stewardship contexts. Their role, therefore, is proposed not as autonomous evaluators but as scalable first-line aid within supervised human-in-the-loop systems, where calibration and expert oversight remain essential safeguards. LLMs could support a human-in-the-loop AMR communication workflow by pre-screening messages, surfacing scores together with the adapted DISCERN anchors, and flagging borderline or inconsistent cases for expert review. Final decisions would remain with human raters, and disagreement between automated and human judgments would trigger secondary review rather than automatic acceptance.

Future studies, also possibly evaluating standardized interaction modalities, are needed to further explore this and other key public health topics.

## Data Availability

The original contributions presented in the study are included in the article/Supplementary material, further inquiries can be directed to the corresponding author.
