# Supplementary material for: LLM-as-a-judge for infection prevention and control and antimicrobial resistance impact: comparing three main LLMs vs. human experts' assessment
Source: Front Public Health. 2026 Jul 14;14:1874389. doi: 10.3389/fpubh.2026.1874389 (PMC13407514; doi:10.3389/fpubh.2026.1874389)
Supplement: Supplementary file 2 [file Supplementary_File_2.docx]

**Supplementary S2.** CHART Checklist Table

| **#** | **CHART checklist item** | **Reported in (section)** |
| --- | --- | --- |
| **Title & Abstract** | | |
| 1a | State that the study is assessing one or more generative AI-driven chatbots for clinical evidence or health advice. | Title |
| 1b | Apply a structured format, if applicable. | Abstract |
| **Introduction** | | |
| 2a | State the scientific background, rationale, and healthcare context for evaluating the generative AI-driven chatbot(s), referencing relevant literature. | Introduction |
| 2b | State the aims and research questions including target audience, intervention, comparator(s), and outcome(s). | Introduction, last paragraph |
| **Methods — Model Identifiers** | | |
| 3a | State the name and version identifier(s) of the generative AI model(s) and chatbot(s) under evaluation, as well as their date of release or last update. | Methods, LLMs' choice and setting |
| 3b | State whether the generative AI model(s) and chatbot(s) are open-source or closed-source/proprietary. | Methods, LLMs' choice and setting |
| **Methods — Model Details** | | |
| 4a | State whether the generative AI model was a base model or a novel base model, tuned model, or fine-tuned model. | Methods, LLMs' choice and setting |
| 4b | If a base model is used, cite its development in sufficient detail to identify the model. | Methods, LLMs' choice and setting |
| 4c | If a novel base model, tuned model, or fine-tuned model is used, describe the pre- and/or post-implementation/deployment data and parameters. | LLMs' choice and setting |
| **Methods — Prompt Engineering** | | |
| 5a | Describe the evolution of study prompt development. | Methods, Prompting strategy |
| 5ai | Describe the sources of prompts. | Methods, Prompting strategy; Suppl. File 1 |
| 5aii | State the number and characteristics of the individual(s) involved in prompt engineering. | Methods, Prompting strategy |
| 5aiii | Provide details of any patient and public involvement during prompt engineering. | Methods, Prompting strategy; Declarations, Ethics approval |
| 5b | Provide study prompts. | Methods, Prompting strategy; Suppl. File 1 |
| **Methods — Query Strategy** | | |
| 6a | State route of access to generative AI model. | Methods, LLMs' choice and setting |
| 6b | State the date(s) and location(s) of queries for the generative AI-driven chatbot(s) including day, month, year, city and country. | Methods, LLMs' choice and setting |
| 6c | Describe whether prompts were input into separate chat session(s). | Methods, Prompting strategy |
| 6d | Provide all generative AI-driven chatbot output/responses. | Availability of data and materials |
| **Methods — Performance Evaluation** | | |
| 7a | Define the ground truth or reference standard used to define successful generative AI-driven chatbot performance. | Methods, Study design |
| 7b | Describe the process undertaken for generative AI-driven chatbot performance evaluation. | Methods, Prompting strategy; Methods, Statistical analysis |
| 7bi | State the number and characteristics of team members involved in performance evaluation. | Methods, Study design (referring to parent study) |
| 7bii | Provide details of any patients and public involvement during the evaluation process. | Declarations, Ethics approval |
| 7biii | State whether evaluators were blinded to the identity of the generative AI-driven chatbot(s) under assessment. | Methods |
| **Methods — Sample Size** | | |
| 8 | Report how the sample size was determined. | Methods, Prompting strategy |
| **Methods — Data Analysis** | | |
| 9a | Describe statistical analysis methods, including any evaluation of reproducibility of generative AI-driven chatbot responses. | Methods, Statistical analysis |
| 9ai | Report the measures used for performance evaluation. | Methods, Statistical analysis |
| **Results** | | |
| 10a | Report the performance evaluation undertaken including the alignment between generative AI-driven chatbot output and ground truth or reference standard. | Results; Tables 1–4 |
| 10b | For responses deviating from the ground truth or reference standard, state the nature of the difference(s). | Results; Discussion |
| 10c | Report the evaluation for potentially harmful, biased, or misleading responses. | Results, last paragraph |
| **Discussion** | | |
| 11a | Interpret study findings in the context of relevant evidence. | Discussion |
| 11b | Describe the strengths and limitations of the study. | Strengths and limitations |
| 11c | Describe the potential implications for practice, education, policy, regulation, and research. | Discussion; Conclusion |
| **Open Science** | | |
| 12a | Report any relevant conflicts of interest for all authors. | Declarations, Competing interests |
| 12b | Report sources of funding and their role in the conduct and reporting of the study. | Declarations, Funding |
| 12c | Describe the process undertaken for ethical approval. | Methods, Ethics consideration; Declarations, Ethics approval |
| 12ci | Describe the measures taken to safeguard data privacy of patient health information, as applicable. | Declarations, Ethics approval |
| 12cii | State whether permission/licensing was obtained for the use of original, copyrighted data. | Declarations, Ethics approval |
| 12d | Provide a study protocol. | Declarations, Availability of data and materials — explicitly states no protocol was registered |
| 12e | State where study data, code repository, and model parameters can be accessed. | Declarations, Availability of data and materials |
