# Supplementary material for: LLM-as-a-judge for infection prevention and control and antimicrobial resistance impact: comparing three main LLMs vs. human experts' assessment
Source: Front Public Health. 2026 Jul 14;14:1874389. doi: 10.3389/fpubh.2026.1874389 (PMC13407514; doi:10.3389/fpubh.2026.1874389)
Supplement: Supplementary file 3 [file Supplementary_File_3.docx]

**Supplementary File 3.** Distribution of Likert ratings assigned by LLMs and human experts stratified by domain

| **Domain** | **Rater** | **Score 1** | **Score 2** | **Score 3** | **Score 4** | **Score 5** |
| --- | --- | --- | --- | --- | --- | --- |
| D1 | Claude | 0 (0.0%) | 0 (0.0%) | 45 (44.6%) | 56 (55.4%) | 0 (0.0%) |
| D1 | Gemini | 0 (0.0%) | 32 (31.7%) | 27 (26.7%) | 42 (41.6%) | 0 (0.0%) |
| D1 | GPT | 1 (1.0%) | 7 (6.9%) | 30 (29.7%) | 60 (59.4%) | 3 (3.0%) |
| D1 | Human | 1 (1.0%) | 27 (26.7%) | 64 (63.4%) | 9 (8.9%) | 0 (0.0%) |
| D2 | Claude | 0 (0.0%) | 1 (1.0%) | 89 (88.1%) | 11 (10.9%) | 0 (0.0%) |
| D2 | Gemini | 0 (0.0%) | 22 (21.8%) | 56 (55.4%) | 23 (22.8%) | 0 (0.0%) |
| D2 | GPT | 1 (1.0%) | 28 (27.7%) | 23 (22.8%) | 47 (46.5%) | 2 (2.0%) |
| D2 | Human | 1 (1.0%) | 14 (13.9%) | 40 (39.6%) | 46 (45.5%) | 0 (0.0%) |
| D3 | Claude | 0 (0.0%) | 0 (0.0%) | 1 (1.0%) | 88 (87.1%) | 12 (11.9%) |
| D3 | Gemini | 0 (0.0%) | 0 (0.0%) | 0 (0.0%) | 85 (84.2%) | 16 (15.8%) |
| D3 | GPT | 5 (5.0%) | 2 (2.0%) | 34 (33.7%) | 60 (59.4%) | 0 (0.0%) |
| D3 | Human | 1 (1.0%) | 4 (4.0%) | 35 (34.7%) | 58 (57.4%) | 3 (3.0%) |
| D4 | Claude | 0 (0.0%) | 14 (13.9%) | 29 (28.7%) | 40 (39.6%) | 18 (17.8%) |
| D4 | Gemini | 0 (0.0%) | 0 (0.0%) | 51 (50.5%) | 16 (15.8%) | 34 (33.7%) |
| D4 | GPT | 0 (0.0%) | 5 (5.0%) | 38 (37.6%) | 37 (36.6%) | 21 (20.8%) |
| D4 | Human | 55 (54.5%) | 16 (15.8%) | 13 (12.9%) | 9 (8.9%) | 8 (7.9%) |
| D5 | Claude | 0 (0.0%) | 1 (1.0%) | 32 (31.7%) | 56 (55.4%) | 12 (11.9%) |
| D5 | Gemini | 0 (0.0%) | 0 (0.0%) | 0 (0.0%) | 39 (38.6%) | 62 (61.4%) |
| D5 | GPT | 0 (0.0%) | 13 (12.9%) | 10 (9.9%) | 30 (29.7%) | 48 (47.5%) |
| D5 | Human | 1 (1.0%) | 9 (8.9%) | 40 (39.6%) | 45 (44.6%) | 6 (5.9%) |

Note: D1: reliability of information, D2: quality of information, D3: persuasiveness, D4: contribution to addressing AMR, D5: overall rating score.
